# Genome-wide association of single nucleotide polymorphism loci and candidate genes for frogeye leaf spot (*Cercospora sojina*) resistance in soybean

**DOI:** 10.1186/s12870-021-03366-y

**Published:** 2021-12-11

**Authors:** Xin Gu, Shanshan Huang, Zhiguo Zhu, Yansong Ma, Xiaohe Yang, Liangliang Yao, Xuedong Gao, Maoming Zhang, Wei Liu, Lei Qiu, Haihong Zhao, Qingsheng Wang, Zengjie Li, Zhimin Li, Qingying Meng, Shuai Yang, Chao Wang, Xiping Hu, Junjie Ding

**Affiliations:** 1 Wuhu Institute of Technology, Wuhu, 241003 China; 2 Jiamusi Branch of Heilongjiang Academy of Agricultural Sciences, Ministry of Agriculture Harmful Biology of Crop Scientific Monitoring Station Jiamusi Experiment Station, China Agriculture Research System of MOF and MARA, Jiamusi, 154007 China; 3 Key Laboratory of Crop Biotechnology Breeding of the Ministry of Agriculture, Beidahuang Kenfeng Seed Co., Ltd., Harbin, 150030 China; 4 Potato Research Institute, Heilongjiang Academy of Agricultural Sciences, Harbin, 150086 China

**Keywords:** Soybean, *Cercospora sojina*, Genome-wide association study, Resistant haplotype, Frogeye leaf spot resistant genes

## Abstract

**Background:**

Frogeye leaf spot (FLS) is a destructive fungal disease that affects soybean production. The most economical and effective strategy to control FLS is the use of resistant cultivars. However, the use of a limited number of resistant loci in FLS management will be countered by the emergence of new high-virulence *Cercospora sojina* races. Therefore, we identified quantitative trait loci (QTL) that control resistance to FLS and identified novel resistant genes using a genome-wide association study (GWAS) on 234 Chinese soybean cultivars.

**Results:**

A total of 30,890 single nucleotide polymorphism (SNP) markers were used to estimate linkage disequilibrium (LD) and population structure. The GWAS results showed four loci (*p* < 0.0001) distributed over chromosomes (Chr.) 5 and 20, that are significantly associated with FLS resistance. No previous studies have reported resistance loci in these regions. Subsequently, 45 genes in the two resistance-related haplotype blocks were annotated. Among them, *Glyma20g31630* encoding pyruvate dehydrogenase (PDH), *Glyma05g28980,* which encodes mitogen-activated protein kinase 7 (MPK7), and *Glyma20g31510*, *Glyma20g31520* encoding calcium-dependent protein kinase 4 (CDPK4) in the haplotype blocks deserves special attention.

**Conclusions:**

This study showed that GWAS can be employed as an effective strategy for identifying disease resistance traits in soybean and narrowing SNPs and candidate genes. The prediction of candidate genes in the haplotype blocks identified by disease resistance loci can provide a useful reference to study systemic disease resistance.

**Supplementary Information:**

The online version contains supplementary material available at 10.1186/s12870-021-03366-y.

## Background

In most soybean-growing countries, soybeans are prone to many plant diseases. Among these, frogeye leaf spot (FLS) caused by the fungus *Cercospora sojina* Hara (*C. sojina*) is one of the most economically harmful [1]. In the main soybean-producing areas of Northeast China [2], the local temperature and leaf wetness periods are very suitable for the occurrence of FLS and epidemics [3]. Because the disease is polycyclic, a suitable environment and improper control measures can result in outbreaks [4]. Although FLS can be controlled by fungicides, there are challenges, such as fungicide resistance and environmental pollution [5]. Thus, the development of resistant cultivars is a preferred disease management strategy. However, the disadvantage of this strategy is that the resistance of cultivars may be rapidly lost. The main reason for this is that resistance mechanisms can be overcome by the emergence of new *C. sojina* pathotypes [6]. Therefore, high-density markers and methods to counter new pathotype races are key to disease resistance breeding.

*Rcs*1 [7] (resistant to *C. sojina* race 1), *Rcs*2 [8], and *Rcs*3 [9] have been recognised by the Soybean Committee [1]. Although the selection pressures on *C. sojina* populations caused by planting resistant cultivars have produced FLS strains that have overcome the *Rcs*1 and *Rcs*2 genes [9], the *Rcs*3 gene continues to confer resistance against most races of *C. sojina* in the U.S.A. [10]. Quantitative trait loci (QTL) were mapped close to a known resistance gene cluster on the soybean linkage group (LG) J by restriction fragment length polymorphism (RFLP) and simple sequence repeats (SSR) [11]. The gene was mapped in the 2-centimorgan (cM) interval between SSR markers Satt244 and Satt547. Several *Phialophora gregata* resistance genes, such as *Rbs*1, *Rbs*2, and *Rbs*3, conferring resistance against brown stem rot, have also been mapped within 10 cM of the *Rcs*3 gene [12, 13]. Two SSR markers associated with *Rcs*3 were confirmed in 64 soybean cultivars and breeding lines of “Davis” ancestors and progenies [14]. However, SSR markers are less suitable for high-throughput and association studies because they are based on repeat length variants, electrophoresis detection methods, and the occurrence of SSR alleles of identical size but different evolutionary origins [15]. Several single nucleotide polymorphisms (SNPs) and insertions or deletion (indels) associated with *Rcs*3 have been evaluated. A total of 19 SNPs/Indels were identified and verified, although only 11 SNPs/Indels were located close to *Rcs*3 in an F_2_ population of “Davis” × “Blackhawk.” The *Rcs*3 gene was located close to Satt244 and 0.50 cM from SNPs AZ573TA150 and AZ573CA393. Neither SNPs nor indels had a direct effect on the phenotype of *Rcs*3; however, 11 SNPs were located in the 3-cM interval around *Rcs*3 [16]. Subsequently, five plant introductions (PIs) from China (PI594619, PI594661, PI594662A, PI594774, and PI594891) were found to be resistant to FLS in a broad soybean spectrum [17]. Bulked segregant analysis (BSA) results showed that the resistance genes in PI594619 and PI594662A were located near Satt501 on chromosome (Chr.) 18 (LG-G), and near Satt547 and Satt244 on Chr. 16 (LG-J), respectively. A resistance gene in PI594661 was found near Satt244 on Chr.16 (LG-J). In addition, the resistance of PI594891 and PI594774 to FLS was controlled by two dominant genes on Chr. 13 near Satt114, which is different from the *Rcs*3 allele on Chr. 18. They had a high resistance level similar to the *Rcs*3 gene in “Davis” among the reported FLS resistance genes [17]. The analysis of lines with key recombination events was used to narrow down the FLS-resistance genomic region of PI594891 from 3.3 Mb to 72.6 kb with five annotated genes. The resistance gene of PI594774 was fine-mapped into a 540-kb region, including 72.6 kb of PI594891. Five candidate genes of Pi594891 were sequenced, and multiple mutations in the promoter, intron, and 5′ and 3′ UTR regions were found [18].

Genome-wide association studies (GWASs) can mine alleles using phenotypic variation and recombination during population evolution. This is significantly related to the variation of target traits at the whole-genome level without constructing a mapping population [19]. GWASs have been widely used to identify resistance genes in soybeans. Che et al. [20] performed a GWAS on a collection of 219 soybean breeding lines for resistance to the SC3 strain of soybean mosaic virus (SMV). A total of 24 SNPs were identified, which accounted for 25.54–33.60% of the phenotypic variation. Many SNPs were found close to *Rsv*1, *Rsv*4, and *Rsv*5. To study soybean resistance to *Sclerotinia* stem rot (SSR) by GWASs, Wei et al. [21] genotyped and sequenced 420 soybean lines. Two computational models (compressed mixed linear model with genome association and prediction integrated tool, fixed and random model circulating probability unification) were used to identify SNPs that were significantly associated with disease response. These similar but also different results indicated that the use of different computational models can affect the SNP association with SSR resistance. A total of 125 genes located in the linkage disequilibrium (LD) of Chr. 1, Chr. 11, and Chr. Eighteen were identified using the two models. The candidate genes in these LD blocks can encode isochorismate synthase and regulate host cell death pathways. GWASs can be used to investigate a set of genetically unstructured genotypes. More precise QTLs can be detected in cases of sufficiently used genetic markers. Vuong [22] studied a set of 553 soybean accessions and genotyped and identified 14 loci distributed on different chromosomes, including 60 SNPs significantly associated with soybean cyst nematode (SCN) resistance. GWAS results also confirmed six QTLs and eight novel QTLs that were previously mapped using bi-parental populations. Many studies have shown that GWASs based on LD can be used to examine the genetic variation in soybean disease resistance traits. It is the main tool to identify candidate resistance genes by associating the phenotypic trait with its underlying genetics [23]. However, there are no reports of GWASs being used to study resistance to *C. sojina* in soybean.

This study analysed 234 soybean accessions using the genotype data of the SoySNP180K BeadChips. We used general linear model and mixed linear model to evaluate the genetic effects of resistance sites and identify resistance-related SNP loci and haplotypes. Possible candidate resistance genes were also annotated. This is an effective approach for molecular breeding and the identification of disease resistance mechanisms.

## Methods

### Plant material and phenotypic evaluation

A total of 234 soybean accessions collected from three different breeding departments in northeast China was used for GWAS. The accessions were public and available for non-commercial purposes. The FLS strain used was *C. sojina* Race15, which was identified as the dominant race among Chinese differential cultivars. It was collected from the Jianan Farm in Jiamusi City (N46°4725.64″ N, E130°305.63″ E). Thereafter, the strain was stored in liquid nitrogen after single spore isolation. All soybean accessions were provided by the Beidahuang Kenfeng Seed Co., Ltd., and *C. sojina* strains were provided by the Jiamusi branch of the Heilongjiang Academy of Agricultural Sciences.

The valuation experiments of FLS incidence were evaluated during the growing season from 2017 to 2019. Before spray inoculation*,* the strain was purified by single-spore isolation and then cultured on V8 juice agar (V8 juice 200 mL/L, CaCO_3_ 3.0 g/L, agar 15.0 g/L, sterile water to 1 L) [24], and gently washed down from the medium using sterile water and filtering through sterilised multi-layer gauze, the conidial suspensions were adjusted to a concentration of 6 × 10^4^ conidia mL^− 1^. At the V2–V3 growth stage, one trifoliate leaf per soybean seedling was inoculated with 0.3 mL of the conidial suspension on the upper leaf surface. Thereafter, the inoculated seedlings were transferred to a humidity chamber at 26–28 °C for 72 h. At 14 and 21 d after inoculation, resistance assessment was evaluated primarily on leaf spot size and the number of spots formed on the most severely affected trifoliate leaves. The disease evaluation scale was as follows: spots ≤2 mm diameter, or ≤ 10 spots of 2–3 mm diameter per trifoliate leaf were categorised as a resistant response (R); 10–20 spots of ≤3 mm diameter per trifoliate leaf were categorised as moderately resistant (MR); > 20 spots of 3 mm were categorised as a susceptible response (S); spots that were connected into large groups and most of the leaves dying early due to the disease were categorised as a highly susceptible response (HS) [1](Additional file 1: Fig. S1). To facilitate the later statistical analysis, the resistance grade of soybean accessions expressed in digits, R is represented by 3, MR, S and HS were 5, 7, and 9, respectively. All inoculation tests were repeated at least three times, and 30 plants of each soybean accession were inoculated. Descriptive statistics of phenotypic data were performed using R software [25].

### Genotyping

DNA from 234 accessions was extracted from the samples using the magnetic bead method. The DNA concentration was determined using a Nanodrop 8000 spectrophotometer (Thermo Fisher Scientific). SNP genotyping of the germplasm population was performed at Beidahuang Kenfeng Seed Co., Ltd., using the SoySNP180K Bead Chip (Thermo Fisher Scientific).

### GWAS analysis and linkage disequilibrium analysis (LD)

The 234 soybean accessions were genotyped using the SoySNP180K BeadChips. Two models, the general linear model (GLM) and the mixed linear model (MLM) correcting for K-matrix (MLM [K]), were used to reduce errors from relative kinship. The threshold of the SNP locus that was significantly associated with the trait was set at *p* < 0.0001. The remaining GWAS analyses were also performed using TASSEL 5.0, including the descriptive analysis, genetic distance matrix of soybean varieties of genotype data, principal component analysis, and kinship matrix [26]. Cluster analysis was performed using the UPGMA method, and a phylogenetic tree was drawn using Mega4 software. The Quantile-Quantile (Q-Q) plots and Manhattan were drawn using TASSEL5.0. The degree of linkage disequilibrium (LD) between each pair of SNPs was estimated with the correlation coefficient *r*^*2*^ using Haploview5.0 (minor allele freq: 0.05. The fraction of strong LD informative comparisons must be at least 0.97, and all other parameters used are default parameters).

### Candidate genes annotation

According to the LD calculated in the previous step, the LD block was determined, and candidate genes were searched in the LD block. Functional annotation of the genes was searched against the NR [27] and GO [28] databases to identify candidate genes related to FLS resistance. Finally, candidate genes were identified by pathway analysis using the Kyoto Encyclopaedia of Genes and Genomes (KEGG) (https://www.kegg.jp/) [29].

## Results

### Phenotyping

We initially analysed the genotypes of 382 soybean accessions using the SoySNP180K BeadChips. A total of 180,961 SNP markers were selected across the 20 soybean chromosomes of cultivated (*Glycine max*) and wild (*G. soja*) soybean accessions. The number of SNPs is estimated to provide approximately 1 SNP every 6.1 kb. Among these, 58,388 of the SNP markers were of the Poly High Resolution type and accounted for 32.24% of the total markers. SNP markers were selected in the next step. After comparing the data over 3 years, 277 accessions with stable results were selected as the candidate population for the GWAS of the resistance genes. Finally, 234 accessions with both genotype and phenotype data were selected as the GWAS population. All the tested accessions had different degrees of lesions, including the most resistant genotypes, indicating that the infection process was successful. There were seven resistant accessions (R), 119 moderately resistant accessions (MR), 87 susceptible accessions (S), and 21 highly susceptible accessions (HS). (Additional file 2: Table S1).

### Genotyping

**Fig. 1:**
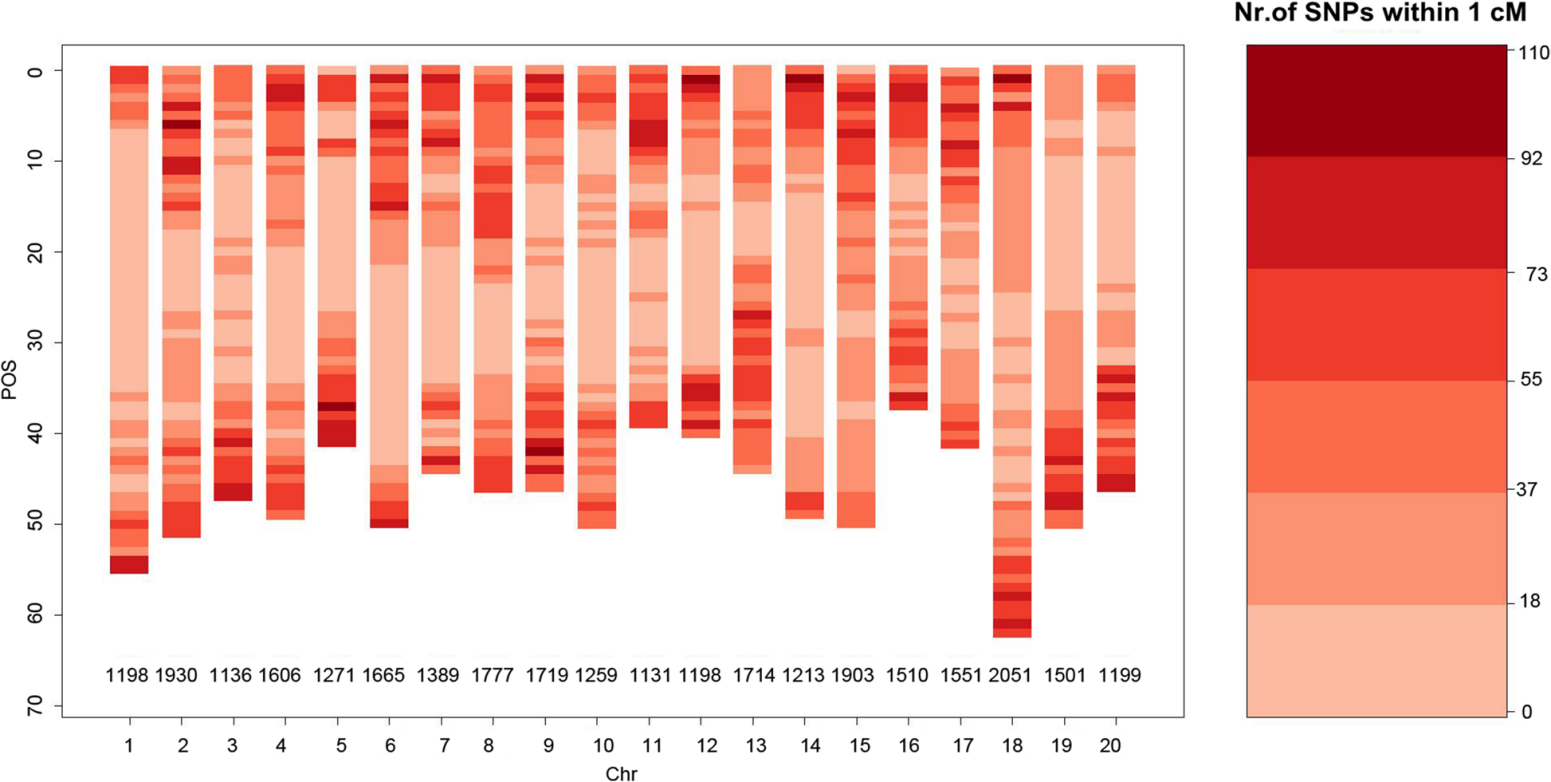
Distribution of the 30,890 SNPs on the 20 soybean chromosomes. The graph shows the number of SNPs with a MAF ≥ 0.05 on each chromosome. The red bar from light to dark on the right represents the number of SNPs within 1 cM

A total of 58,388 SNP genotypes from 234 accessions were analysed using descriptive analysis. The results showed that 27,302 SNPs had no polymorphism in the association analysis population. After filtering, 30,890 SNPs on 20 soybean chromosomes were selected as genotype data sources for GWAS. The number of SNPs on Chr.18 was the largest (2051), and the number of SNPs on Chr. 1 and Chr.12 had the least (1198) (Fig. 1). On average, there were 1544.5 SNPs on each chromosome, and each SNP covered 31.301 kb of the chromosome (Table 1).

**Table 1:** Distribution of 30,890 SNPs on 20 chromosomes of soybean

Chromosome	No. of SNP markers	Starting position (kb)	End position (kb)	SNP coverage (kb)	Single SNP coverage (kb/SNP)
1	1198	46.209	55,901.759	55,855.55	46.624
2	1930	59.603	51,642.224	51,582.621	26.727
3	1436	18.316	47,723.675	47,705.359	33.221
4	1606	30.661	49,194.977	49,164.316	30.613
5	1274	69.827	41,932.26	41,862.433	32.859
6	1665	10.299	50,630.237	50,619.938	30.402
7	1389	7.129	44,605.694	44,598.565	32.108
8	1777	57.34	46,925.494	46,868.154	26.375
9	1719	18.921	46,832.712	46,813.791	27.233
10	1259	98.244	50,913.805	50,815.561	40.362
11	1434	16.255	39,163.086	39,146.831	27.299
12	1198	46.664	40,103.384	40,056.72	33.436
13	1714	9.149	44,339.594	44,330.445	25.864
14	1243	14.558	49,706.317	49,691.759	39.977
15	1903	9.353	50,860.462	50,851.109	26.722
16	1540	17.629	37,327.774	37,310.145	24.227
17	1551	252.274	41,878.591	41,626.317	26.838
18	2051	7.193	62,264.999	62,257.806	30.355
19	1504	18.1	50,558.55	50,540.45	33.604
20	1499	31.395	46,769.259	46,737.864	31.179

### Population structure analysis of 234 accessions

**Fig. 2:**
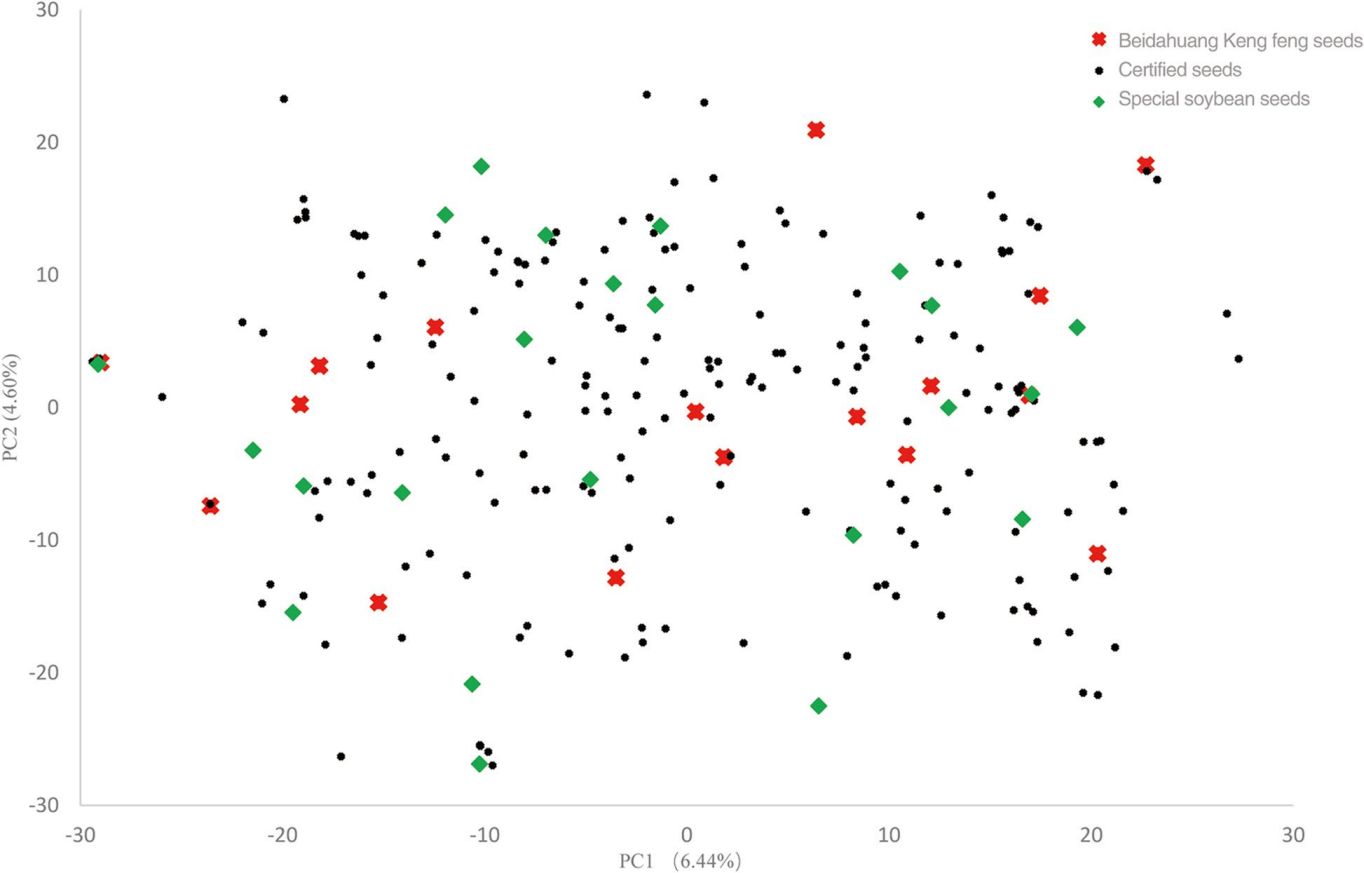
Principal Component Analysis (PCA) of a diverse set of 243 soybean cultivars. PCA scatter plot showing the two main principal components. Dots with different colors and shapes represent different sources. Ken Feng seeds means that soybean accessions come from Beidahuang Keng feng seed Co., Ltd., Certified seeds means that accessions was approved by the Soybean Certification Committee of Heilongjiang Province, Special soybean seeds means that accessions are used for special purposes, such as black coat soybean, brown coat soybean, adzuki soybean and fresh special soybean

A total of 30,890 SNPs from 234 accessions were analysed using principal component analysis (PCA). The first and second principal components explained 6.44 and 4.60% of the variance, respectively, and explained 11.04% of the phenotypic variation. A scatter plot of the first and second principal components showed that the soybean genotypes collected from different sources were closer to each other. A subpopulation structure was not observed in this population (Fig. 2). Cluster analysis of 234 soybean accessions based on UPGMA was conducted using 30,890 SNP marker genotypes. There was no obvious classification of the accessions, consistent with the PCA results (Additional file 3: Fig. S2).

### GWAS of genes resistant to *C. sojina*

**Fig. 3:**
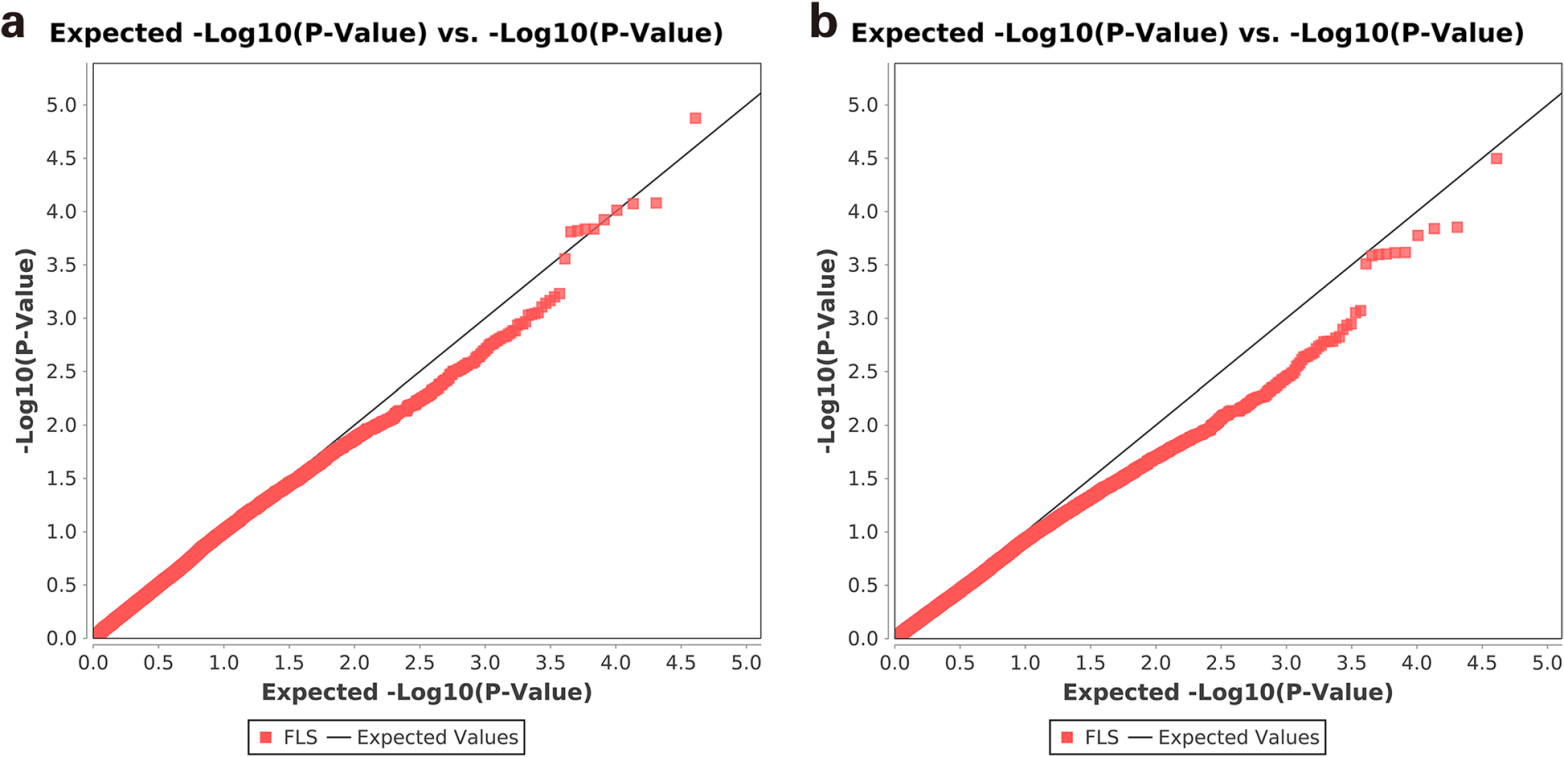
Quantile-quantile plots for frogeye leaf spot resistance using two models. **a** General linear model. **b** Mixed linear model

**Fig. 4:**
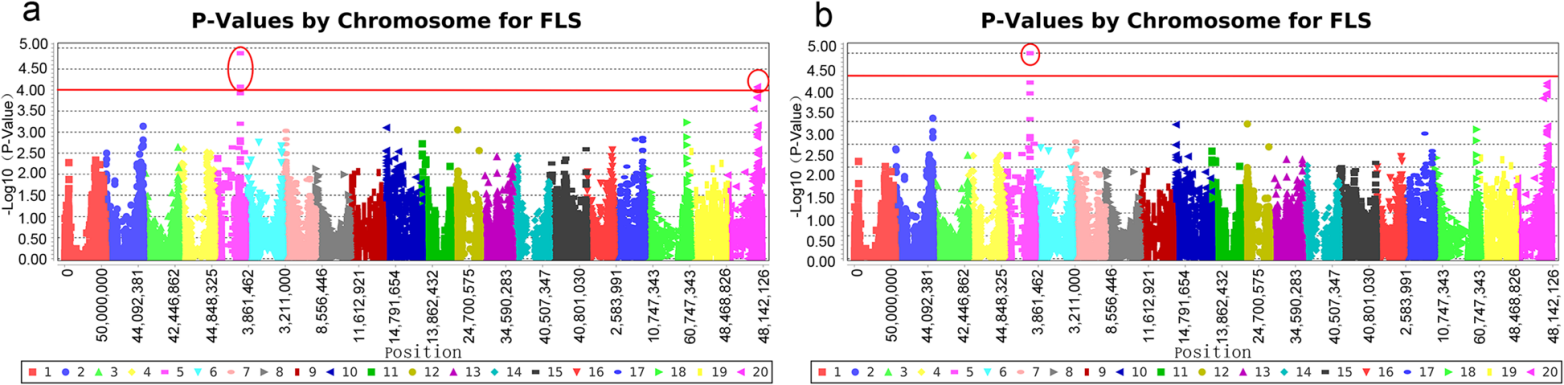
Manhattan plots of the association of SNPs with FLS resistance in soybean identified by GWAS. Chromosomal SNPs can be differentiated by various colours. The red dashed line represents the significance threshold, −log_10_ (p) = 3.00. **a** Manhattan plot for resistance by General linear model. **b** Manhattan plot for resistance by Mixed linear model

GLM and MLM models for GWAS were evaluated, and the degree of consistency between the observed and expected *p-*values was assessed using QQ plots. Both the models controlled the generation of false positives well (Fig. 3), and the significant SNPs associated with disease resistance traits were displayed on Manhattan plots (Fig. 4). In total, four SNPs that were significantly associated with disease resistance traits were detected on Chr. 5 and Chr. 20, respectively, according to both GLM and MLM analyses (Table 2).

**Table 2:** SNP loci significantly associated with *C. sojina* resistance traits by GWAS

Methods	Marker	Chromosome	Position	Marker F	***p*** value	Add F	Add p	Dom F	Dom p
GLM	Affx-89,062,122	5	34,658,974	11.79327	1.33E-05	0.21198	0.64566	23.35405	2.46E-06
	Affx-89,220,750	5	34,687,389	9.78979	8.31E-05	0.04618	0.83004	19.5297	1.53E-05
	Affx-89,210,591	20	40,270,311	9.77378	8.43E-05	2.56054	0.11093	16.81175	5.73E-05
	Affx-89,163,218	20	40,262,553	9.62294	9.71E-05	2.82916	0.09392	16.22938	7.64E-05
MLM(K)	Affx-89,062,122	5	34,658,974	10.83494	3.19E-05	0.11133	0.73894	21.4606	6.05E-06

### Haplotype analysis for FLS resistance gene

**Fig. 5:**
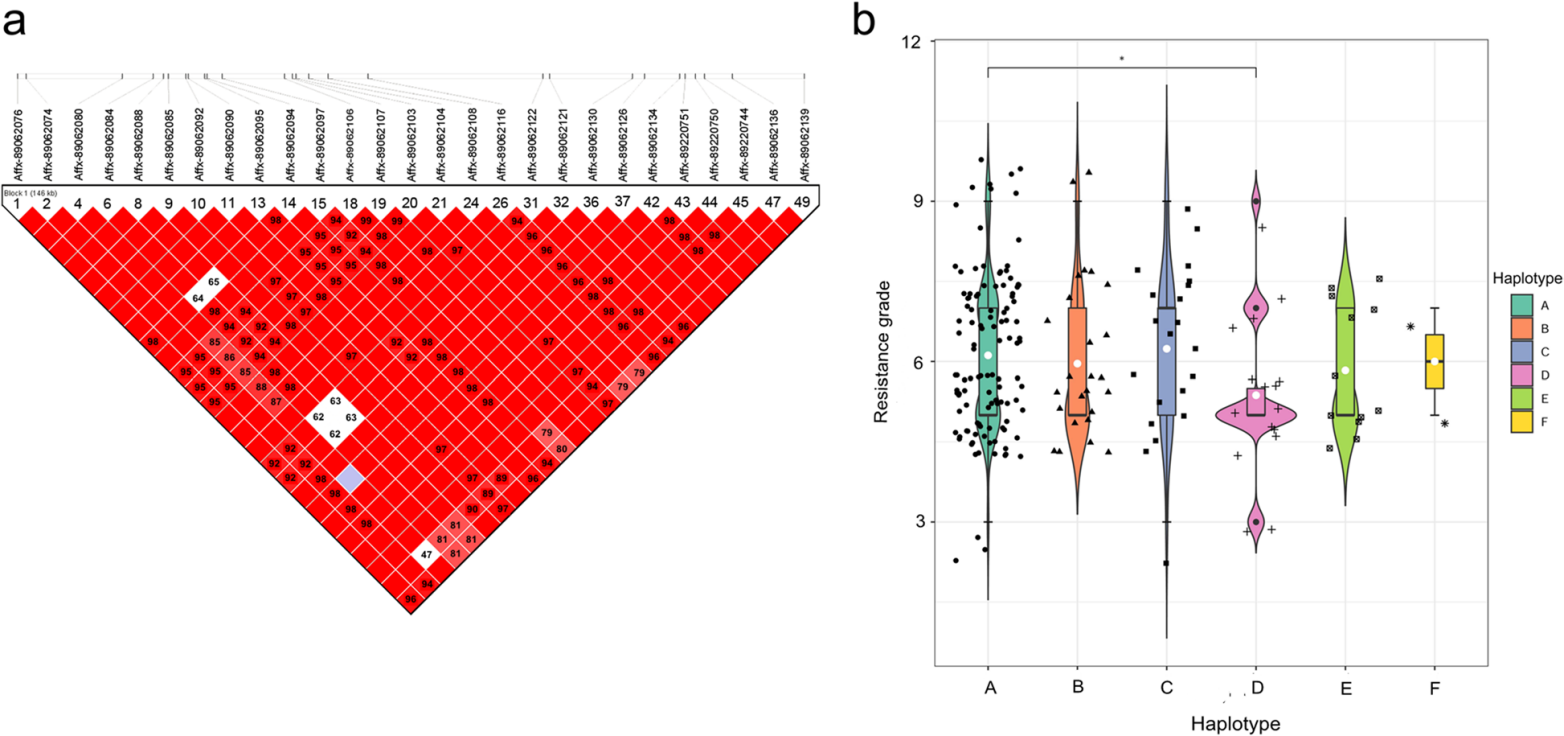
Haplotype block for SNPs significantly associated with FLS resistance on Chr. 5. **a** Numbers in squares indicate 100-fold *r*^2^ values of each pair of SNPs. The bars above LD plots represent the physical positions of SNPs. LD blocks are marked with black triangles. **b** Box plots showing the resistance grade and the distribution of six haplotypes in soybean accessions. The score of the Y axis on the left is the resistance grade of Soybean accessions expressed in digits, resistant response is represented by 3, moderately resistant response is represented by 5, susceptible response is represented by 7, and highly susceptible response is represented by 9. ‘*‘refers to a significant difference in the resistance grade among the haplotypes (*p* < 0.05)

**Fig. 6:**
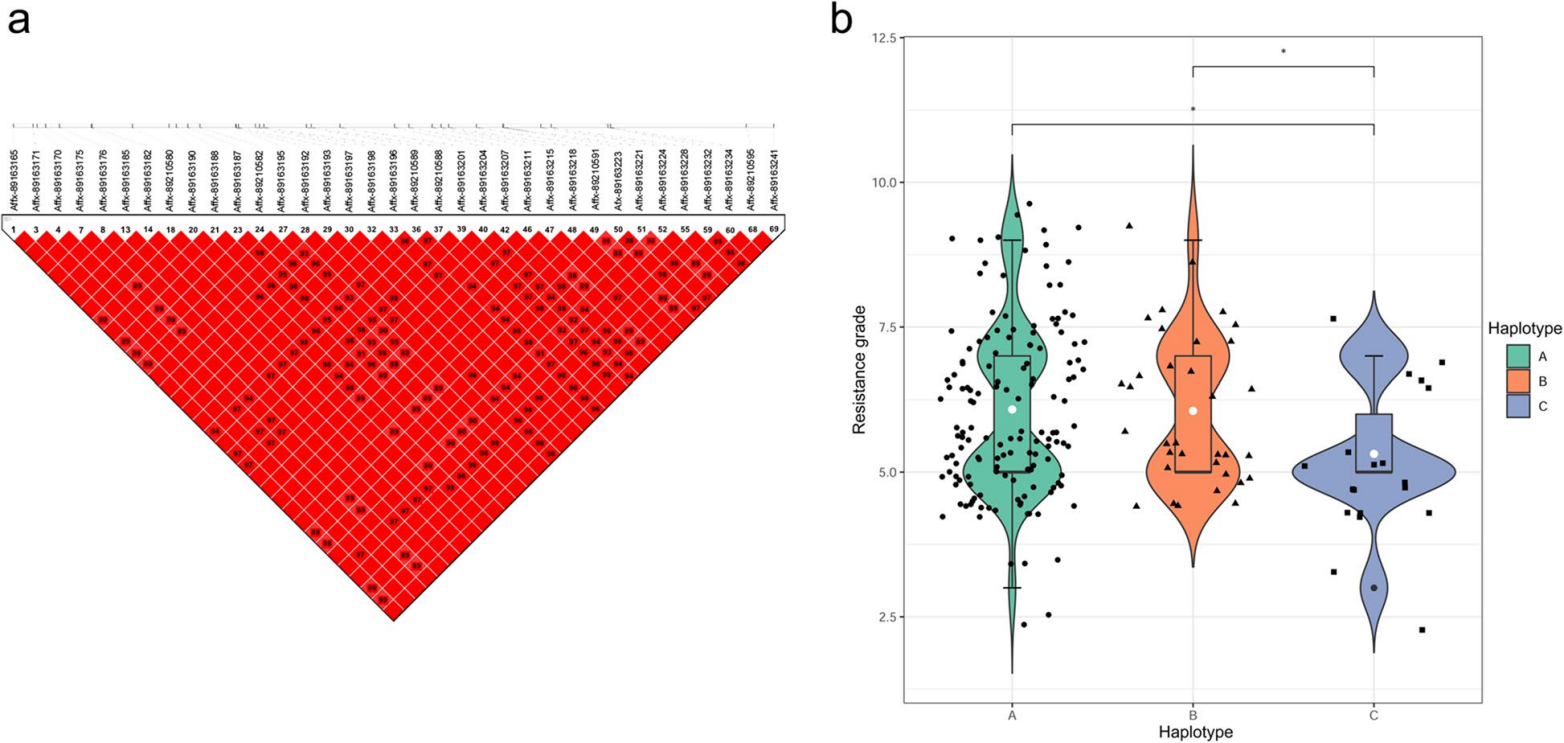
Haplotype block for SNPs significantly associated with FLS resistance on Chr. 20. **a** Numbers in squares indicate 100-fold *r*^2^ values of each pair of SNPs. The bars above LD plots represent the physical positions of SNPs. LD blocks are marked with black triangles. **b** Box plots showing the resistance grade and the distribution of three haplotypes in soybean accessions. The score of the Y axis on the left is the resistance grade of Soybean accessions expressed in digits, resistant response is represented by 3, moderately resistant response is represented by 5, susceptible response is represented by 7, and highly susceptible response is represented by 9. ‘*‘refers to a significant difference in the resistance grade among the haplotypes (*p* < 0.05)

**Fig. 7:**
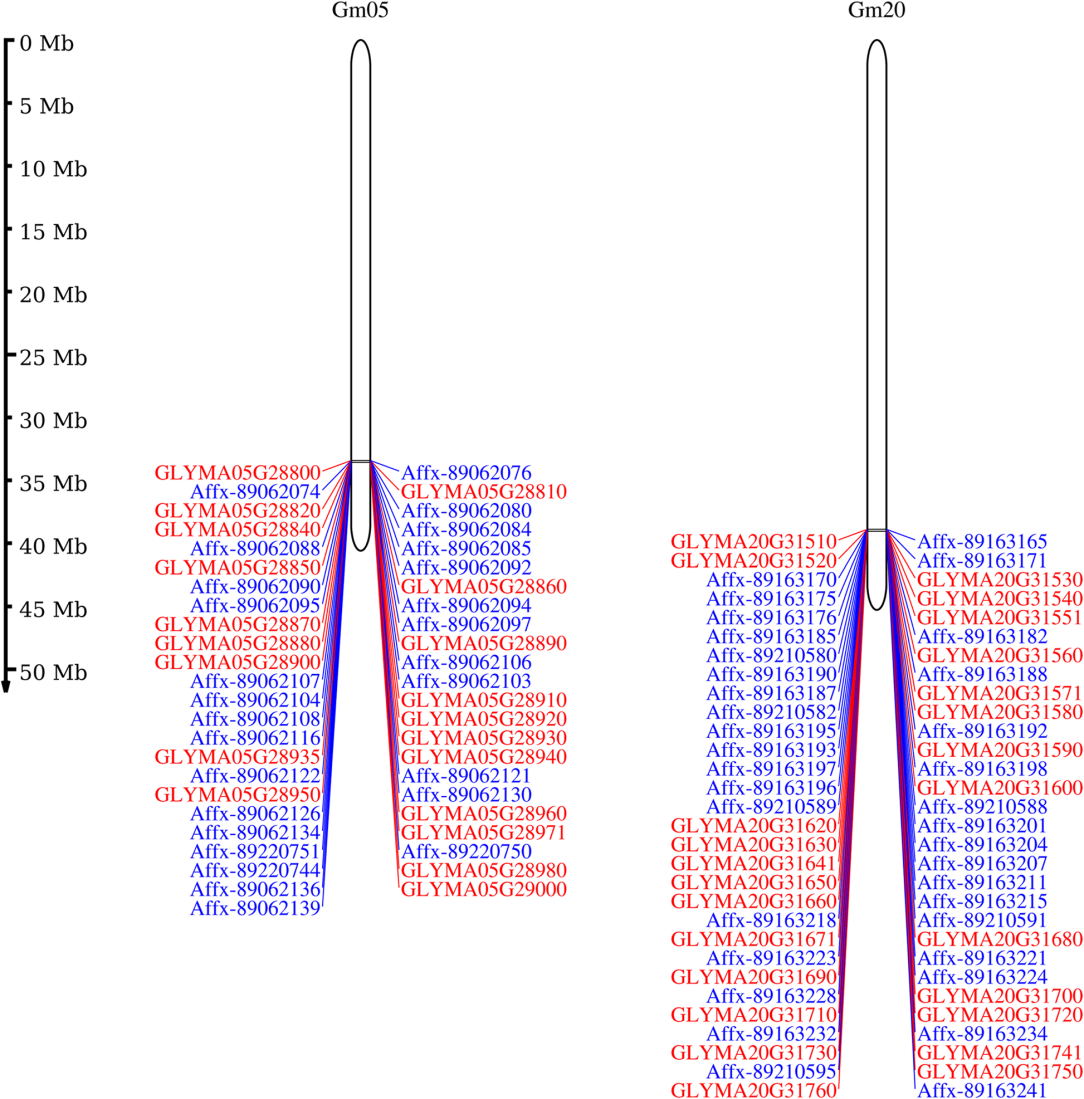
Location map of the gene information in the two haplotype blocks on chromosomes 5 and 20

Linkage disequilibrium (LD) between pairs of SNPs on Chr. 5, and Chr. 20 was analysed using the Haploview Software 5.0. *C. sojina* resistance-related SNPs and adjacent SNPs formed different haplotype blocks. A total of 27 SNPs on Chr. 5 were located in one adjacent haplotype block, forming six haplotypes (Additional file 4: Table S2) (Fig. 5a). A total of 35 SNPs on Chr. 20 were located in one adjacent haplotype block, forming three haplotypes (Additional file 4: Table S2) (Fig. 6a). On Chr. 5, Hap A was significantly more resistant to FLS than Hap D (*p* = 0.025, *p* < 0.05). Hap A is the resistant genotype, and Hap D is the susceptible genotype (Fig. 5b). On Chr. 20, Hap A was significantly more resistant to FLS than Hap C (*p* = 0.016, *p* < 0.05). Hap B was significantly more resistant to FLS than Hap C (*p* = 0.019, *p* < 0.05). Hap A and Hap B are the resistance genotypes, and Hap C is the susceptible genotype (Fig. 6b). The gene information distributed in the three haplotype blocks was extracted, and the positions on the chromosomes were indicated (Fig. 7).

### Candidate genes for FLS resistance at GWAS loci

A total of 45 genes within the two haplotype blocks of Chr. 5 and Chr. 20 were annotated with Glyma1.0 in the NR, GO, and KEGG databases (Additional file 5: Table S3). These genes were separated into 29 GO terms, including mitochondrial outer membrane (GO:0005741), calcium-dependent protein serine/threonine kinase activity (GO:0009931), calcium-dependent protein kinase activity (GO:0010857), MAP kinase activity (GO:0004707), protoxylem development (GO:0090059), and xylan metabolic process (GO:0045491) (Additional file 6: Table S4). The enriched KEGG pathway is involved in plant–pathogen interaction (gmx04626), MAPK signalling pathway–plant interaction (gmx04016), and biosynthesis of secondary metabolites (gmx01110) (Additional file 7: Table S5). Among these genes, *Glyma05g28980* encodes mitogen-activated protein kinase 7 (MPK7). *Glyma20g31510* and *Glyma20g31520* encode calcium-dependent protein kinase (CDPK4) family proteins, and *Glyma20g31630* encodes pyruvate dehydrogenase (PDH), which may be involved in plant disease resistance. These genes were predicted to be candidate resistance genes.

## Discussion

GWASs have made significant progress in soybean genetics research; however, this study is limited to a few characters [30]. Compared with traditional QTL mapping, whole-genome association analysis has the advantages of a wide detection range [31], high resolution [32, 33], and more material sources [34]. However, the GWAS analysis has limitations. Association mapping is complementary to traditional bi-parental linkage mapping; however, bi-parental linkage mapping cannot be replaced. The interaction effects of genes and the environment will affect GWAS analysis results; therefore, rigorous phenotyping is required. Due to the scale of the study, more complex traits controlled by multiple loci with relatively small phenotypic effects will require large populations [35]. Population structure is a significant factor in correlation studies; therefore, it is necessary to select germplasm resources and evaluate the population structure carefully. This study used more than three replications to increase the accuracy of phenotype identification. In addition, GWAS is only a prediction of candidate genetic sites, and further research is needed in combination with other experimental methods to explore their biological functions.

Lee [36] reduced the number of SNPs related to target traits by adjusting *p*-value selectively. Only the most significant SNP in each LD block was selected as the representative location and listed by trait, environment, and analytical method. Selecting a low threshold *p*-value increases the possibility of false positives. In the present study, we selected two models for association analysis and considered the number of SNP loci associated with disease resistance traits, when *p* < 0.0001 was selected as the threshold. In contrast, the GLM was found slightly better than the MLM. However, this study shows relatively less statistical power, therefore we retained the two models assuming to obtain more reliable correlation sites. Although the statistical power was improved, we found that most of the observed significance was still lower than expected and therefore, the results of this study are considered to have relatively weak statistical power. This may be due to the linkage disequilibrium between a large number of SNP loci in the population, and the number of significant loci (loci without linkage disequilibrium) is significantly lower than the actual number of loci, and the expected p-value is underestimated. However, a small number of loci reached the threshold. Therefore, these loci remain very important and need to be verified.

We obtained SNP sites at the physical positions Gm5:34561008–34,707,609, Gm20:40140684–40,353,461. Lin et al. [37] used QTL mapping and GWAS to identify loci conferring partial resistance to *Pythium sylvaticum* in soybean. They found that a significant SNP marker overlapped with the QTL identified for partial resistance to *Pythium sylvaticum* at Gm20:2245263. Hu et al. [38] studied the resistance loci of pod dehiscence and Jing et al. [39] to the soybean sclerotinia stem rot by GWAS, and found that the resistance loci were located at Gm20:8202869 and Gm20:33803317, respectively. Both of these loci found were significantly away from the loci found in the present study. Che et al. [20] used genome-wide association to study soybean mosaic virus SC3 resistance and found four SNP sites located in Gm20:41544070–41,680,482, which were also confirmed to be related to disease resistance in anti-SCN (soybean cyst nematode) studies. Sara et al. [40] used a diverse panel to reveal the genetic architecture of charcoal rot (*Macrophomina phaseolina*) resistance in soybean and found the resistance loci at Gm20:42356434, which are very close to the distance found in this study.

*C. sojina* races used in previous sequencing studies are unique to the US, and we used the latest variant race of China. Different countries adopt their own local differential soybean cultivars to identify *C. sojina* races by phenotype, making the races unable to be unified [6]. Genetic studies of many plant-pathogen interactions indicate that plants often contain single locus that confers resistance to specific races of a pathogen containing a complementary avirulence gene [41]. Race-specific interactions in the FLS of soybeans have been shown to follow a gene-for-gene model. Sharma et al. detected some additional minor FLS resistance loci in LG A1 (Chr.5) and I (Chr.20) [5]. Among these loci, one was associated with Satt440 on LG I (Chr. 20) at 112.7 cM. The locus explained 15% of the variation in FLS at 42 dpi (days after manual infestation) (*p* = 0.001) with the “Essex” allele reducing disease severity by up to 0.95 units [42]. However, its genetic distance cannot be converted, therefore it cannot be compared with this study. When the selected loci were compared with the known loci, no coverage was found. It is inferred that the four SNPs associated with FLS resistance may represent new loci that require further verification.

In this study, one encoding the PDH gene, one encoding the MPK7 gene, and two encoding CDPK4 genes in the haplotype block were worthy of attention. They are all related to the biological pathway of salicylic acid (SA). The plant systemic acquired resistance (SAR) is an inducible immune system. The cells in the infected parts of plants can produce signal molecules such as salicylic acid (SA), lipids, peptides, and nitric oxide. These signal molecules diffuse to the normal tissues and cells of plants through the vascular system and then activate the expression of stress resistance genes and the regulation of physiological metabolism in normal cells, thereby enhancing the immune ability of cells and effectively restricting disease spread [43]. SA can induce the expression of various pathogenesis-related (PR) genes and help plants resist the invasion of disease organisms, such as viruses, bacteria, and fungi. SA is involved in the formation of plant innate immunity and effector-triggered immunity in plants. SA can also activate the suicide process of plant cells and form necrotic spots in the infected parts to prevent the invasion and spread of pathogens [44]. The gene encoding MPK7 (GhMPK7) cloned from cotton belongs to the C-MAPK group. It plays an important role in broad-spectrum resistance to fungi and viruses regulated by SA and is also involved in the regulation of plant growth and development [45]. MPK7 is co-expressed with MKK3 and promotes strong expression of *Pseudomonas syringae* resistance genes in plants [46]. In addition, this study proved that the MKK3 pathway plays a role in pathogen defence and further underscores the importance and complexity of MAPK signalling in plant stress responses. MKK3 plays a role in jasmonate (JA)-mediated developmental signalling and generates H_2_O_2_ to activate MPK7, which acts as a secondary signal to activate defence genes [47]. Among phytohormones, JA plays an important role in resisting biological stress [48]. However, reverse genetic studies have indicated that MAPKs, SA-induced protein kinase (SIPK), and wound-induced protein kinase (WIPK), are rapidly activated by fatty acid–amino acid conjugates. MAPKs and calcium-dependent protein kinase (CDPK) are necessary for the induction of JA in response to biological stress [49]. When CDPK4 and CDPK5 in *Nicotiana attenuata* were simultaneously silenced, transgenic plants (IRcdpk4/5) induced high levels of defense metabolites. A study by Yang [49] found that CDPK4 and CDPK5 affect plant resistance to biological stress in a JA- and JA-signalling-dependent manner. Transgenic plants showed over-activation of SIPK, a MAPK involved in various stress responses, and genetic analysis indicated that the increased SIPK activity in IRcdpk4/5 plants leads to exceptionally high JA levels. Some studies have found that CPK4/11 and CPK5/6 play an important role in the resistance of *Arabidopsis* to *Pseudomonas syringae* in a MAPK-independent manner [50]. The effector in *Heterodera avenae* can interact specifically with an Arabidopsis pyruvate dehydrogenase subunit, which might interfere with the SA signalling pathway and suppress plant defence responses [50]. We identified candidate genes for soybean resistance to FLS using an association analysis [17]. The effective use of these QTLs requires functional verification combined with proteomics when effective markers are identified for use in resistance breeding.

## Conclusion

Based on the GWAS results of the GLM and MLM models, a total of four SNPs were associated with FLS resistance, of which were located on Chr. 5 and Chr. 20, respectively. Resistance-related SNPs and adjacent SNPs formed two haplotype blocks. Then, 45 candidate genes in the haplotype blocks were annotated in the NR, GO, and KEGG databases. Four of these are worthy of special attention, these proteins are directly or indirectly involved in the biological pathway of salicylic acid (SA) and jasmonic acid (JA). These two plant hormones may induce the expression of disease resistance-related genes and are essential for plant systemic acquired resistance (SAR). Our study provides useful information on the underlying mechanisms of FLS resistance.

## Supplementary Information

**Additional file 1: Figure S1.** Scale of disease resistance grade

**Additional file 2: Table S1.** Phenotypic evaluation of 234 soybean accessions

**Additional file 3: Figure S2.** Dendrogram analysis of 234 soybean accessions by UPGMA

**Additional file 4: Table S2.** Resistance-related SNPs and adjacent SNPs formed two haplotype blocks on chromosomes 5 and 20

**Additional file 5: Table S3.** Functional classification of genes in two haplotype blocks on chromosomes 5 and 20

**Additional file 6: Table S4.** GO enrichment analysis of genes in the two haplotype blocks

**Additional file 7: Table S5.** KEGG pathway analysis of genes in the two haplotype blocks

## Data Availability

Data generated or analysed during this study are included in this published article and its supplementary information files.

## Abbreviations

GWAS: Genome-wide association analysis
FLS: Frogeye leaf spot
QTL: Quantitative trait loci
SNP: Single nucleotide polymorphism
LD: Linkage disequilibrium
PCA: Principal component analysis
MLM: Mixed linear model
