# Supplementary figures and images for: Genome-wide association of single nucleotide polymorphism loci and candidate genes for frogeye leaf spot (Cercospora sojina) resistance in soybean
Source: BMC Plant Biol. 2021 Dec 11;21:588. doi: 10.1186/s12870-021-03366-y (PMC8665500; doi:10.1186/s12870-021-03366-y)

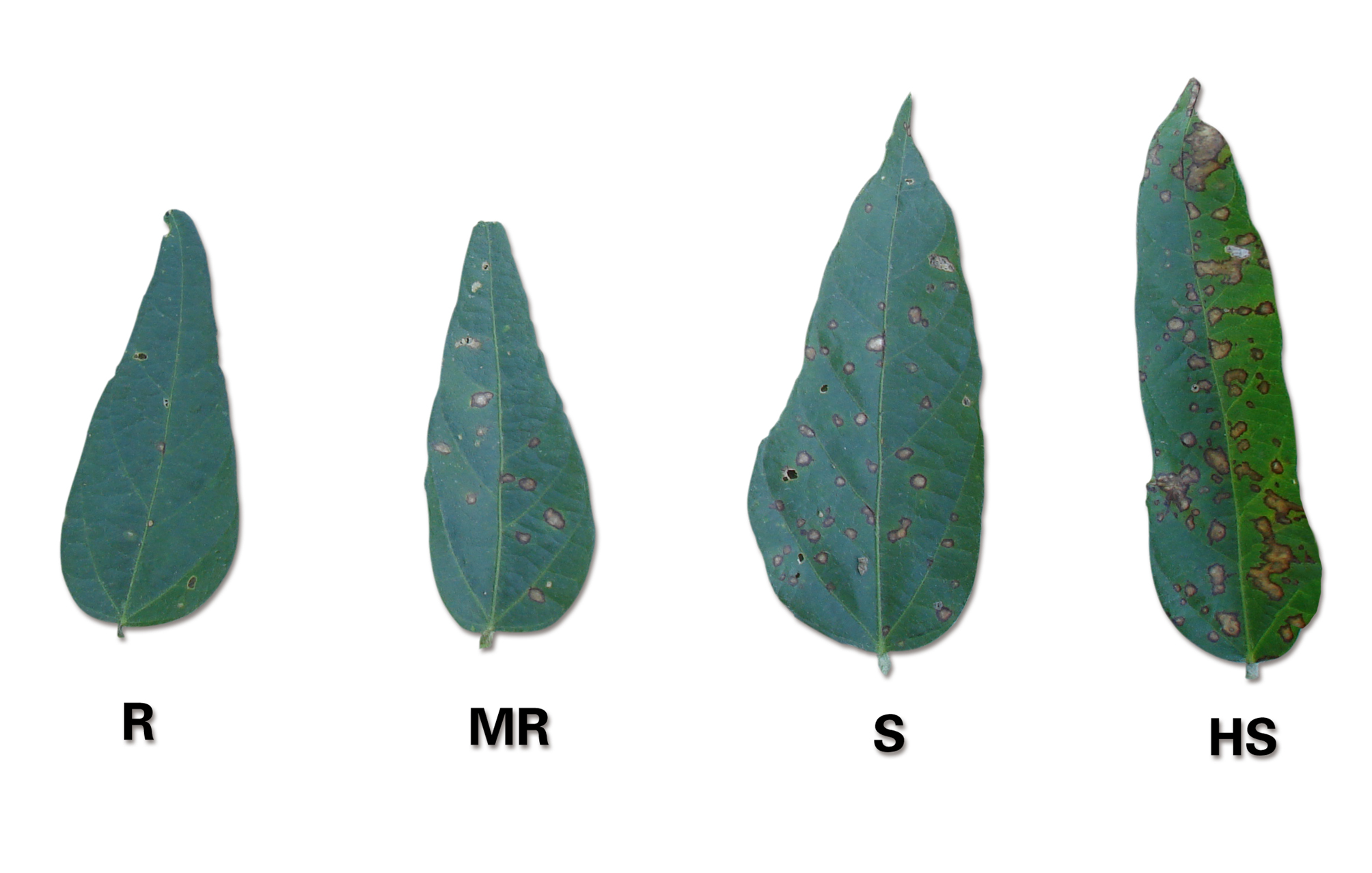

Supplement: Supplementary file 1 — Additional file 1: Figure S1. Scale of disease resistance grade [file 12870_2021_3366_MOESM1_ESM.jpg]

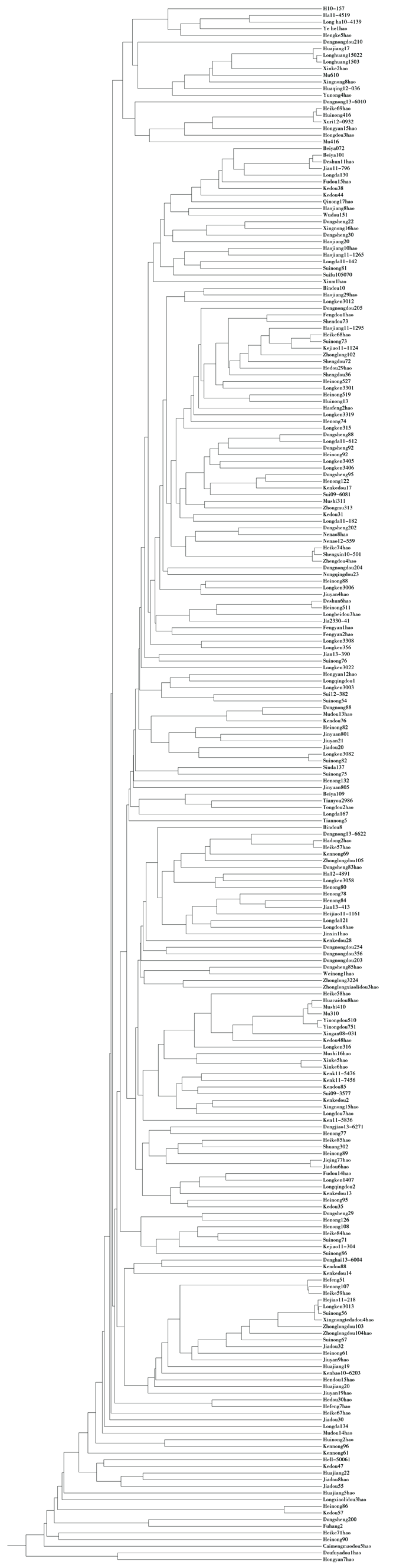

Supplement: Supplementary file 3 — Additional file 3: Figure S2. Dendrogram analysis of 234 soybean accessions by UPGMA [file 12870_2021_3366_MOESM3_ESM.tif]
